# The PI3K inhibitor GDC-0941 displays promising *in vitro and in vivo* efficacy for targeted medulloblastoma therapy

**DOI:** 10.18632/oncotarget.2742

**Published:** 2014-12-06

**Authors:** Michael Ehrhardt, Rogerio B. Craveiro, Martin I. Holst, Torsten Pietsch, Dagmar Dilloo

**Affiliations:** ^1^ Department of Pediatric Hematology and Oncology, Center for Pediatrics, University of Bonn Medical Center, D-53113 Bonn, Germany; ^2^ Department of Neuropathology, University of Bonn, 53105 Bonn, Germany

**Keywords:** medulloblastoma, GDC-0941, PI3K inhibitor, targeted therapy, c-myc

## Abstract

Deregulation of the Phosphoinositide 3-kinase (PI3K)/AKT signalling network is a hallmark of oncogenesis. Also medulloblastoma, the most common malignant brain tumor in children, is characterized by high levels of AKT phosphorylation and activated PI3K signalling in medulloblastoma is associated with enhanced cellular motility, survival and chemoresistency underscoring its role of as a potential therapeutic target. Here we demonstrate that GDC-0941, a highly specific PI3K inhibitor with good clinical tolerability and promising anti-neoplastic activity in adult cancer, also displays anti-proliferative and pro-apoptotic effects in pediatric human medulloblastoma cell lines. Loss in cell viability is accompanied by reduced phosphorylation of AKT, a downstream target of PI3K. Furthermore, we show that GDC-0941 attenuates the migratory capacity of medulloblastoma cells and targets subpopulations expressing the stem cell marker CD133. GDC-0941 also synergizes with the standard medulloblastoma chemotherapeutic etoposide. In an orthotopic xenograft model of the most aggressive human medulloblastoma variant we document that oral adminstration of GDC-0941 impairs tumor growth and significantly prolongs survival. These findings provide a rational to further investigate GDC-0941 alone and in combination with standard chemotherapeutics for medulloblastoma treatment.

## INTRODUCTION

Medulloblastoma is the most common malignant brain tumor in children. Current medulloblastoma therapy comprises surgery, chemotherapy and radiotherapy in older children. Despite remarkably advances over the past decades, long-term survival still range from 40% to 70% [1]. Moreover, survivors often suffer from long-term treatment related neurological sequelae [2]. Thus novel more specific therapies are urgently needed.

Medulloblastoma can be divided into 5 histological and at least four biological subgroups – WNT, SHH, group 3 and group 4 - with distinct cellular origins, genetic changes, gene-expression -methylation profiles as well as clinical course [3, 4]. In cancerogenesis the Phosphoinositide 3-kinase (PI3K)/AKT signalling pathway has been identified as a key driver of cellular proliferation, migration and angiogenesis. This also pertains to human medulloblastoma in which activation of PI3K/AKT signaling has been linked to enhanced tumor growth, metastasis and chemoresistancy [5–7]. Indeed, we and others have previously shown that primary medulloblastoma of different genetic variants display high levels of AKT phosphorylation [5–7]. Also PTEN, a negative regulator of the PI3K/AKT pathway, is frequently downregulated by promoter hypermethylation and/or allelic losses of the chromosomal region 10q23 [5]. As a target for cancer therapy PI3K/AKT is of particular interest as it serves as an integration node in a network of tumor-promoting signalling pathways including receptor tyrosine kinases. In medulloblastoma, increased copy number of the platelet-derived growth factor receptor alpha (PDGFRα) and overexpression of the epidermal growth factor receptor (EGFR) are associated with poor outcome [8] [9]. Also, inhibition of tyrosine receptor-activation suppresses cancer-promoting functions *in vitro* and *in vivo* [10] [11].

Inspite of the pivotal role of PI3K/AKT in human medulloblastoma, a xenograft model delineating *in vivo* the anti-proliferative and pro-apoptotic effects of PI3K inhibition is currently lacking. This is in part due to the fact that first generation PI3K inhibitors such as wortmannin, LY294002 and PIK-75 are encumbered by high *in vivo* toxicity, and broad off-target activity. [12–17]. Thus until recently, efforts to further validate PI3K inhibition for treatment of medulloblastoma have been thwarted by lack of pharmaceutical inhibitors suitable for patient use. Taking advantage of the novel, highly specific, clinical grade Pan-PI3K inhibitor GDC-0941 [18], we confirm that PI3K/AKT signalling is indeed a critical target for anti-cancer therapy in human medulloblastoma. In adult cancer GDC-0941 has been well-tolerated and exhibited promising anti-neoplastic activity in phase I/Ib trials of ovarian breast, non small cell lung cancer and multiple myeloma [19–22].

Here we present first evidence that in human medulloblastoma, GDC-0941 inhibits proliferation, induces apoptosis and acts synergistic with etoposide in suppressing medulloblastoma cell viability. Moreover, GDC-0941-mediated PI3K-inhibition reduces the clonogenicity of medulloblastoma cells and leads to significant reduction of CD133 expressing stem cell-like medulloblastoma subpopulations. Most importantly we demonstrate that in an orthotopic xenograft model of the most aggressive *c-myc*-amplified human medulloblastoma variant GDC-0941 adminstration result in tumor growth delay and survival benefit underscoring the potential of PI3K-inhibition for medulloblastoma therapy.

## RESULTS

To analyse the potential of GDC-0941 in pediatric medulloblastoma cell lines we have used a panel of human medulloblastoma cell lines of which MEB-Med-8A, D283 Med, D341 exhibit distinct characteristics of the clinically most aggressive genetic group 3 variant. The fourth cell line Daoy is derived from TP53-mutated desmoplastic medulloblastoma and displaying markers of SHH-group tumors [23–30].

### GDC-0941 treatment leads to profound reduction of cell viability

**Figure 1:**
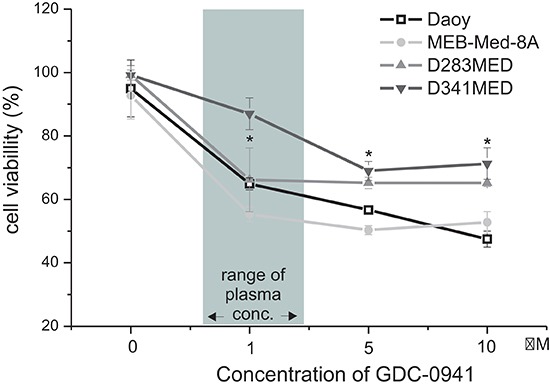
GDC-0941 treatment leads to a dose-dependent reduction of medulloblastoma cell viability GDC-0941 decreased the cell viability of the established medulloblastoma cell lines MEB-Med-8A, D283 Med, Daoy and D341 Med. The cell lines were treated with increasing concentrations of GDC-0941. Area shaded in gray indicates the range of GDC-0941 concentration detected in patient's levels. The vehicle DMSO served as control. After 48 h of drug exposure the cell viability was assessed by means of the MTS assay. Data points below asterisks differ significantly (*p < 0.05) from the control. Each experiment was performed in triplicates and repeated four times.

In a dose-response study the cytotoxic capacity of the PI3K inhibitor GDC-0941 was evaluated in the medulloblastoma cell lines by MTS assay at 48 h under standard growth conditions (Figure 1). The vehicle DMSO served as control. The dose range chosen for *in vitro* and *in vivo* studies was based on pharmakokinetic data available form phase I/II studies as detailed in the discussion. At GDC-concentrations corresponding to patient plasma levels (1–2 μM) cell viability was substantially reduced in three of four medulloblastoma cell lines, namely to 64.9% ± 1.9% in Daoy, 55.3 ± 2.3% in MEB-Med-8A and 66.2% ± 10% D283 Med with a negligible accentuation of the observed inhibitory effect in the presence of a ten fold higher GDC-0941 dose. In contrast at 1 μM GDC-0941, D341 Med was largely unaffected with cell viability maintained as high as 89.9 ± 4.5%. Yet, when escalating the drug dose by a log, a decrease in cell viability to 71.3 ± 5.0% was achieved.

### GDC-0941 displays anti-proliferative and pro-apoptotic effects in medulloblastoma cells

**Figure 2:**
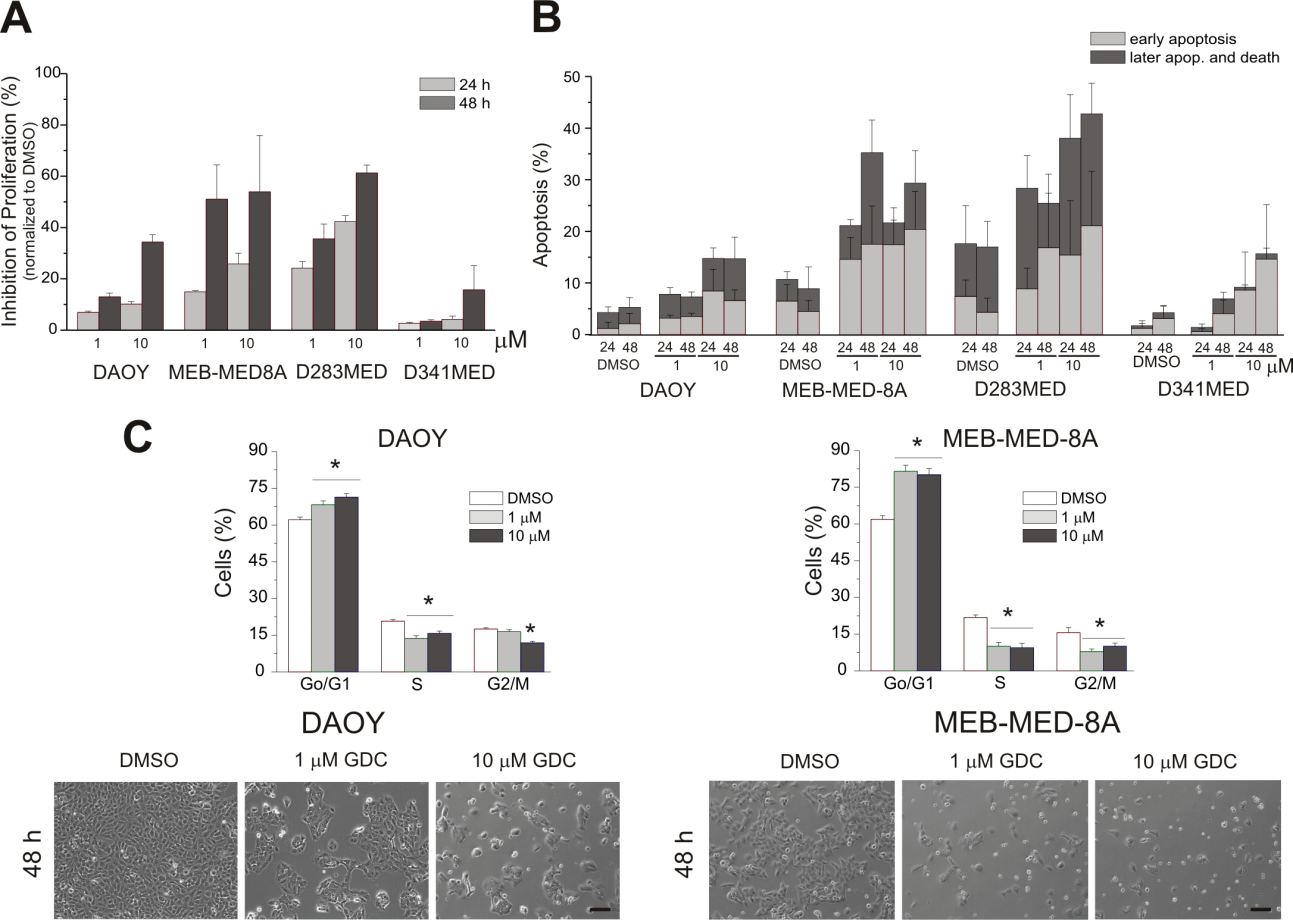
Determination of anti-proliferative and pro-apoptotic effects of GDC-0941 in medulloblastoma cells In a combined proliferation-apoptosis assay based on a CFSE-7AAD-Annexin-V staining the capacity of GDC-0941 to inhibit proliferation **(A)** and induce apoptosis **(B)** in the stated medulloblastoma cell lines was determined. The cells were treated with 1 μM and 10 μM of GDC-0941 for 24 and 48 h respectively. The vehicle DMSO served as control. In contrast to apoptosis, proliferation inhibition was normalized to the control DMSO. All stated average values for proliferation inhibition and apoptosis induction differed significantly (*p* < 0.05) from the DMSO control except the value for induction of apoptosis at 1 μM for the cell line D341 Med. The data represents four independent experiments. **(C)** upper panel - GDC-0941 induces a G1-phase cell cycle arrest. Daoy and MEB-Med8A cells were exposed to 1 and 10 μM of GDC-0941 for 48 h. Subsequently the cell cycle distribution was determined by Hoechst 33342 staining. The vehicle DMSO served as control. The lower panel in Figure 2C visualizes the effect of GDC-0941 on the adherent cell lines Daoy and MEB-Med-8A. Statistically significant differences are marked by an asterisk (*p < 0.05). The data shown represents four independent experiments. The reduction in cell density and change in cell morphology is depicted. Scale bar 100 μm.

With the intent to further dissect the observed loss in cell viability into anti-proliferative and pro-apoptotic effects, we performed a flow cytometry-based combined CFSE-7AAD-Annexin-V assay (Figure 2). After 48 h of culture at the clinically relevant concentration of 1 μM GDC-0941, cellular growth of MEB-Med-8A and D283 Med cells was significantly reduced with 51.1 ± 10.4% and 35.6 ± 5.8% inhibition of proliferation respectively. Daoy cells proved themselves more GDC-resistant with 13.0 ± 1.47% inhibition of proliferation, while D341 Med cells were essentially unaffected. Escalating the GDC-dose to 10 μM enhanced the attenuating effect on proliferation to 34.3 ± 2.9% in Daoy and 15.7 ± 9.4% in D341 Med cells. GDC-0941 also induced apoptosis in all four medulloblastoma cell lines at a concentration corresponding to patient plasma levels (Figure 2B). Thus after 48 h at 1 μM GDC-0941, 35.5 ± 9% of MEB-Med-8A and 37.2 ± 10% of D283 Med cells were apoptotic. In Daoy and D341 cells apoptosis rates were below 5%, which could be enhanced to 16.7 ± 9% for Daoy when escalating the drug dose by a log.

### Treatment of medulloblastoma with GDC-0941 induces G0/G1-phase cell cycle arrest

PI3K activity is known to be involved in cell cycle progression [31, 32]. Because of their differential response profile to GDC-0941 treatment, MEB-Med-8A and Daoy were chosen to assess cell cycle distribution after 48 h of drug treatment (Figure 2C, upper panel). Here we demonstrate that PI3K inhibition arrests medulloblastoma cells at the G0/G1-Phase checkpoint. (MEB-Med-8A G0/G1-Phase: DMSO 65.7% ± 1.9%, GDC-0941 1 μM 86.2% ± 1.1%; Daoy G0/G1-Phase: DMSO 64.4% ± 0.9%, 1μM 73.8% ± 1.4%).

### The anti-proliferative and pro-apoptotic effects of GDC-0941 are associated with inhibition of the PI3K/AKT pathway

**Figure 3:**
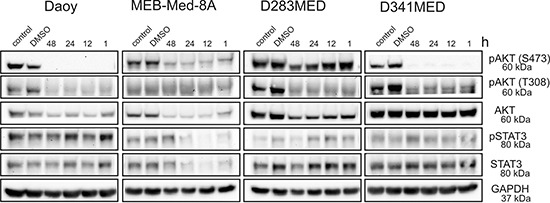
The anti-proliferative and pro-apoptotic effects of GDC-0941 are associated with a reduction of AKT phosphorylation Daoy, MEB-Med-8A, D283 Med and D341 Med cells were treated with 1 μM of GDC-0941 for 1, 12, 24 and 48 h. Total protein levels and the phosphorylation status of AKT^T308/S473^ and STAT3^Y705^ were determined by Western blot. GAPDH served as loading control.

Expression and constitutive activation of AKT, a downstream target of the PI3K has been delineated in primary medulloblastoma tumour samples [5–7], In accordance with these findings, we show that the four investigated medulloblastoma cell lines were phosphorylated at the catalytic sites T308 and S473 of AKT (Figure 3). Of note, treatment with 1 μM GDC-0941 led to marked reduction of AKT^T308/S473^ phosphorylation in all four medulloblastoma cell lines as early as 1 hour after drug exposure. Indeed AKT-phorphorylation was even completely abrogated in D341 Med cells. Yet, only in the three medulloblastoma lines MEB-Med-8A and D283 Med and Daoy most responsive to GDC-mediated cytotoxic effects, AKT protein synthesis itself was compromised in addition to impaired phosphorylation.

Aberrant activation of the transcription factor STAT3 is considered critical for medulloblastoma pathogenesis. In keeping with these findings the four investigated medulloblastoma cell lines display phosphorylation of Tyr705 of STAT3 (Figure 3). A crosstalk between the PI3K/AKT pathway and STAT3 signaling network has recently been suggested [33] and indeed MEB-Med-8A exhibit a reduction of STAT3 phosphorylation and STAT3 protein levels upon GDC-0941 treatment, while in the other medulloblastoma cell lines this interaction was not observed.

### GDC-0941 treatment inhibits medulloblastoma cell migration

**Figure 4:**
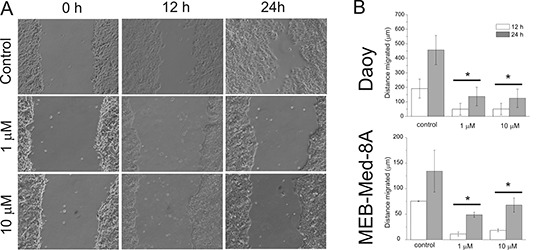
GDC-0941 inhibits medulloblastoma cell migration After a single scratch was made in a confluent monolayer of Daoy and MEB-Med-8A cells, these were exposed to 1 and 10 μM of GDC-0941. Each scratch was photographed after 12 and 24 h and the width of the scratch was determined. **(A)** schematic represention of Daoy cells **(B)** analysis of both cell lines. Statistically significant differences are marked by an asterisk (*p < 0.05). The data shown represent five independent experiments.

Next we determined the potential of GDC-0941 to inhibit tumour cell mobility in a standard scratch wound assay (Figure 4). We chose the cell lines Daoy and MEB-Med-8A because of their known migratory properties. At 1 μM GDC-0941 inhibited cellular migration with 137 ± 63 μm in comparison to 456 ± 100 μm in the Daoy control. MEB-Med-8A although characterized by slower migratory capacity still exhibits a signficant difference in migration of 11 ± 4 μm at 24 h of drug exposure in comparison to 75 ± 1 μm in the respective control. Escalating the dose 10 times did not lead to a further reduction of the migratory potential neither in Daoy nor in MEB-Med-8A cells.

### GDC-0941 reduces the clonogenicity of medulloblastoma cell lines

**Figure 5:**
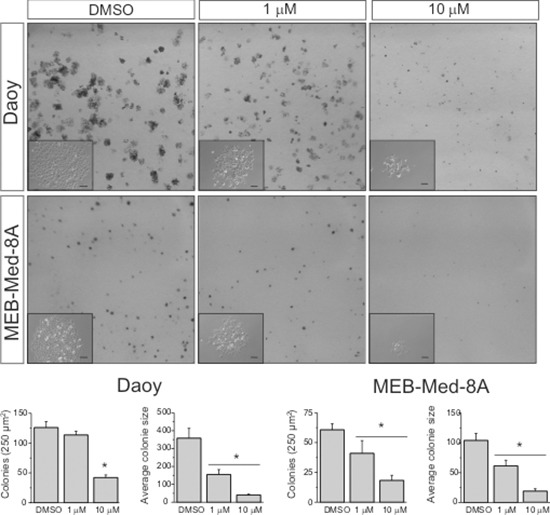
GDC-0941 impairs colony formation of medulloblastoma cells Daoy and MEB-Med-8A cells were exposed to 1 and 10 μM of GDC-0941 for 48 h. Subsequently the cells were maintained in standard growth medium for 6 days, colony formation and colony size were assessed. Statistically significant differences are marked by an asterisk (*p < 0.05). The data shown represent five independent experiments.

We also analyzed the capability of GDC-0941 to interfere with clonogenicity of the adherent medulloblastoma cell lines MEB-Med-8A and Daoy (Figure 5). Treatment of MEB-Med-8A with 1 μM GDC-0941 resulted in a reduction of colony numbers (NC: 41 ± 10) and colony size (ACS: 61 ± 9p^2^) in comparison to control (NC: 60 ± 5; ACS: 104 ± 12p^2^). Also in Daoy, the colony size (155 ± 27p^2^) was significantly smaller than in the control (358 ± 55p^2^) whilst colony numbers were maintained (113 ± 6 vs 126 ± 10). Escalating the concentration of GDC-0941 to 10 μM enhanced the described effects on colony number and size in both cell clines (MEB-Med-8A: NC: 18 ± 4, ACS: 52 ± 8p^2^; Daoy: NC: 42 ± 4, ACS: 40 ± 5p^2^).

### GDC-0941 delays medulloblastoma growth and prolongs survival in an orthotopic medulloblastoma xenograft model

**Figure 6:**
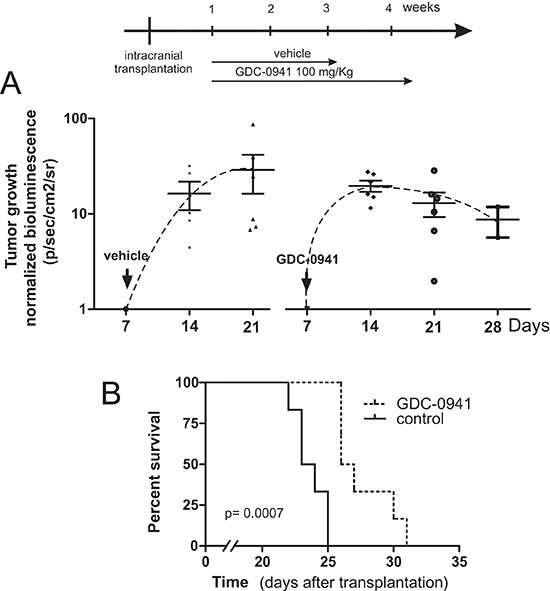
GDC-0941 inhibits tumour growth *in vivo* and prolongs the event-free survival of mice bearing intercranial medulloblastomas In a orthotopic xenograft mouse model we analyzed whether GDC-0941 could inhibit medulloblastoma growth *in vivo*. For this purpose 2 × 10^4^ MEB-Med-8A cells were transplanted into the cerebellum. The mice analyzed for tumor growth by bioluminescent imagining at 1, 2, 3 and 4 weeks. One week after transplantation mice were treated with 100mg/kg of GDC-0941 once daily until symptoms occurred. **(A)** depicts the normalized tumor growth delay while **(B)** shows the survival of treated and untreated animals via Kaplan-Meier plot. GDC-0941 treatment prolonged the symptom-free survival of medulloblastoma bearing mice significantly. The data shown represent six independent experiments.

To assess *in vivo* efficacy of GDC-0941, an orthotopic medulloblastoma model was established (Figure 6). To this end 2 × 10^4^ lentivirally transduced MEB-Med-8A cells stably expressing luciferase were injected into the cerebellum of immunocompromised mice resulting in reliable tumor formation as early as one week post transplantation. Animals with established tumors received 100 mg/kg GDC-0941 or the pharmaceutical vehicle once daily. Tumor growth was monitored by bioluminescence, and mice were taken from the experiment when clinical impairment due to tumor progression was observed. Animals treated with GDC-0941 displayed delayed tumor growth that resulted in significantly longer survival (26.5 days; median) compared to control animals (23.5 days).

## DISCUSSION

Medulloblastoma is the most common malignant pediatric brain tumor that is often resistant to standard therapy. Therefore novel treatment approaches intercepting critical regulatory pathways in cancer development and progression are warranted. Here we present first *in vitro* and *in vivo* evidence that the clinically available pan-PI3K inhibitor GDC-0941 displays potent anti-neoplastic activity in medulloblastoma and commends itself as a possible adjunct to current medulloblastoma treatment.

Indeed in an orthotopic murine model of pre-established human medulloblastoma, treatment with GDC-0941 results in significant survival benefit. Humanized orthotopic models are key to evaluate tumor growth and drug efficacy in the context of a tumor-specific micro-environment. This is particularly true in brain tumors in which biodistribution of the drug is of additional interest. Thus to allow for recovery of the blood barrier, we delayed GDC-treatment for 1 week after medulloblastoma-installation. Such “tumor growth delay” studies also mimic the clinical situation of pre-established tumors more adequately and are of stronger evidence in drug testing than less stringent “tumor inhibition” studies based on concomitant tumor and drug inoculation. [34]. Still we observed significant impairment of tumor-growth after two weeks of GDC-therapy and provide first proof that treatment with the pan PI3K-inhibitor GDC-0941 shows anti-neoplastic efficacy in an orthotopic humanized medulloblastoma model even at a relatively low GDC-dose compared to reports in other tumors [35]. For *in vivo* assessment of GDC-efficacy we chose MEB-Med-8A, a patient-derived human medulloblastoma line that based on its molecular and genetic characteristics corresponds to the most aggressive medulloblastoma group 3, which is characterized by *c-myc* amplification and isochromosome 17. MEB-Med-8a derived tumors also mimic the clinical presentation of this medulloblastoma variant by rapid and invasive growth that leads to animal death within 2–3 weeks [23, 30]. Despite aggressive tumor growth inside the spatially confined fossa cranii posterior housing cerebellum and brainstem, which control coordination of movement, balance, equilibrium and critical vital functions, oral administration of 100 mg/kg GDC-0941 corresponding to intermediate doses applied to patients in phase Ib studies [19, 20] resulted in suppressed medulloblastoma growth and significantly prolonged the survival of treated animals. Yet, for critical appraisal of delayed tumor progression after oral application of PI3K inhibitors, one also needs to consider anti-angiogenic activity as one of the potential underlying mechanisms. Further studies are clearly warranted to dissect the *in vivo* effects of GDC-0941 treatment on PI3K-activity or its downstream elements in the tumormass itsself versus the microtumorenvironment.

Nonetheless, in our extensive *in vitro* analysis we authenticate that exposure to 1 μM GDC-0941 corresponding to patient plasma levels significantly diminishes viable cell numbers in three of four medulloblastoma lines to 50–65%. Only in D341 previously shown to be resistant to selected cytotoxic drugs 5 μM GDC-0941 were required to achieve this level of suppression [36, 37]. We based our choice of *in vitro* drug concentrations on the original publication characterizing GDC-0941 as a potent and selective inhibitor of class I PI3K kinase. In solid tumor cell lines including glioblastoma, cell proliferation was inhibited at IC50s of 0.28–0.95 μM [18] which corresponds to the reported GDC-0941 plasma levels in clinical phase I studies [19, 20]. In these dose escalation trials in patients with solid tumors, single doses of 15 to 600 mg GDC-0941 were applied. Plasma levels at doses not associated with grade 3/4 toxicites ranged from 1–10 μM during the first 12 h. Our *in vitro* assessment reveals that GDC-0941 exerts its profound cytotoxic activity against medulloblastoma at concentrations within the reported patients' plasma range with little additional efficacy upon dose increase even by a log.

Of note, at these dose levels GDC-0941 also sensitizes medulloblastoma lines to etoposite resulting in synergistic impairment of cell viability (Supplementary Figure 1). Moreover, GDC-0941 also compromises the clonogenicity of medulloblastoma cells and targets CD133-positive stem cell-like medulloblastoma subpopulations (Supplementary Figure 2). Further dissection of the anti-neoplastic activity revealed that GDC-0941 not only arrests the proliferation of medulloblastoma cell lines in G0/G1 phase but also induces marked cell death. Beyond these anti-proliferative and pro-apoptotic effects GDC-0941 inhibits medulloblastoma cell migration a pre-requirement for invasion and metastasis. Our results corroborate most recent findings indicating that beyond *c-myc*-amplified medulloblastoma, PI3K inhibition also synergizes with hedgehog pathway inhibition and restrains the growth of SHH-group tumors in a murine allograft medulloblastoma model [27, 38]. Therefore we hypothesize that loss of PTEN or activation of AKT could potentially serve as a future biomarker for the incorporation of GDC-0941 into the standard treatment regimes for pediatric medulloblastoma.

For validation of GDC-capacity to inhibit the PI3K/AKT signaling pathway, the phosphorylation status of the most prominent PI3K downstream signaling molecule, AKT, was selected as a valid marker for PI3K activity [14]. In line with previous reports documenting high activity of the PI3K/AKT pathway in primary medulloblastoma, our data document AKT expression and phosphorylation of the sites T308 and S473 respectively in all four medulloblastoma cell lines analyzed [5–7]. Phosphorylation of these two sites regulates AKT activity and is critical for the oncogenic potency of the protein [39, 40]. In our experiments GDC-mediated anti-neoplastic effects were associated with a reduction in AKT phosphorylation in all investigated cell lines. Thus, in medulloblastoma we delineate the capacity of GDC-0941 to inhibit intracellular signal transduction cascades and underscore the current concept that PI3K/AKT signaling plays a key role in medulloblastoma proliferation, survival and migration [5–7]. Furthermore, prolonged exposure to the PI3K-inhibitor revealed that apart from posttranslational modifications such as reduced AKT phosphorylation, AKT protein levels were suppressed in the highly GDC-0941-sensitive cell lines, Daoy, MEB-Med-8A and D283 Med. This observation is in line with the role of AKT as a main regulator of protein expression via the mTOR signaling complex and might explain the higher susceptibility of these cell lines compared to D341 Med [41, 42].

In various cancer entities including medulloblastoma, the transcription factor STAT3 as downstream target of various receptor tyrosine kinases has been implicated in the regulation of cellular programs including cell-cycle progression, apoptosis, tumour angiogenesis and tumour-cell evasion of the immune system [43–47]. In accordance with these findings the four tested medulloblastoma cell lines are characterized by high STAT3 protein expression and marked phosphorylation of tyrosine 705. Recent proteomic data uncovered a crosstalk of the PI3K pathway and the STAT3 signalling network and ascribed STAT3 a key role in PI3K-driven oncogenic transformation [33]. Here we show that this interaction might also be of relevance in some medulloblastoma. Indeed in MEB-Meb-8A, the most susceptible cell line to GDC-0941-mediated cytotoxicity, PI3K-inhibition led to concomitant loss of STAT3 expression and phosphorylation. In view of future clinical application however, it is noteworthy that in the three other cell lines, STAT3 phosphorylation seems to be independent of PI3K signalling. Thus, targeting PI3K in combination with pathways signalling via STAT3 might enhance the anti-neoplastic activity and prove therapeutical interest, Yet, whether PI3K/STAT3 crosstalk is a common event in medulloblastoma needs to be further elucidated.

In summary, we showed that inhibition of the PI3K signaling cascade by GDC-0941 inhibits medulloblastoma proliferation profoundly, induces significant cell death at concentrations corresponding to patient plasma levels and synergizes with a standard chemotherapeutic applied in pediatric medulloblastoma. Moreover, GDC-0941 reduces CD133-positive stem cell-like subpopulations and compromises the clonogenicity of medulloblastoma cells. Beyond the anti-proliferative and pro-apoptotic effects, GDC-0941 displays marked inhibition of medulloblastoma cell migration, a pre-requisite for tumor invasion. Most importantly, oral adminstration of GDC-0941 delays tumor growth and promotes survival in an orthotopic model of the most aggressive human medulloblastoma variant characterized by *c-myc* amplification. Yet, we hypothesize that benefit of PI3K-inhibitor treatment is not restricted to *c-myc*-amplified medulloblatoma. WNT-, SHH- or group 4 tumors that lack *c-myc* amplification but display PI3K/AKT pathway activation might as well benefit from PI3K inhibition. Thus our data provide a rationale to further evaluate GDC-0941 in combination with standard chemotherapy for the treatment of medulloblastoma.

## MATERIAL AND METHODS

### Reagents and antibodies

GDC-0941 was obtained from LC Laboratories, while etoposide (VP16) and Hoechst were provided by Sigma. Primary antibodies - pSTAT3 (Tyr705, D3A7), STAT3 (124H6), pAKT (Thr308, 244F9), pAKT (Ser473), AKT (11E7) and GAPDH were purchased from Cell Signalling while secondary antibodies were purchased from Dianova. Carboxyfluoreszein-Succinimidyl-Ester (CFSE) was obtained from Invitrogen, D-Luciferin sodium salt was purchased from PJK.

### Animals

Immunocompromised (NOD/SCID IL2Rγ^Null^ (NSG) mice were obtained from Charles Rivers.

### Cell culture

The human medulloblastoma cell lines Daoy (HTB 186), D283 Med (HTB185) and D341 Med (HTB-187) were obtained from American Type Culture Collection. The medulloblastoma cell line MEB-Med-8A was generated by us from a large cell medulloblastoma (TP). The medulloblastoma cell lines Daoy, D283 Med and MEB-Med-8A were maintained in complete medium, namely Dulbecco's Modified Eagle Medium (DMEM, Invitrogen) with L-glutamine supplemented with 1 mM sodium pyruvate (PAA), 1% penicillin/streptomycin (Invitrogen) and 10% fetal bovine serum (FBS, Invitrogen). The medulloblastoma cell line D341 Med was maintained in DMEM with L-glutamine supplemented with 1mM sodium pyruvate, 1% penicillin/streptomycin and 10% human serum (PAA).

### Cell viability assay

Cell viability was assessed with CellTiter 96 Aqueous One Solution Cell proliferation Assay (Promega) which contains 3-(4,5-dimethylthiazol-2-yl)-5-(3-carboxymethoxyphenyl)-2-(4-sulfophenyl)-2H-tetrazolium (MTS). To ensure a linear growth curve over 48 h for assessment of GDC-0941 inhibitor mediated effects, each well of 96-well plates was seeded with 2.5 × 10^3^ Daoy, 6 × 10^3^ MEB-Med-8A, 10^4^ D283 Med and 10^4^ D341 Med cells, respectively. After overnight culture in complete medium, the cells were treated with increasing GDC-0941 concentrations. The vehicle dimethylsulfoxide (DMSO) served as control. After 48 h MTS was added according to the supplier's protocol and the absorbance was measured at 490 nm using an ELISA plate reader (Victor^2^ Wallac, Perkin Elmer). Cell viability was calculated in percent of control.

### Combined cell proliferation and apoptosis assay

Medulloblastoma cells were stained with CFSE according to the supplier's instructions. Daoy (3 × 10^5^/6 well), MEB-Med-8A (5 × 10^5^/well), D283 Med (5 × 10^5^/well) and D341 Med (5 × 10^5^/well) cells were seeded in 6-well cell culture dishes in complete medium. After overnight culture, the cells were treated with different concentrations of GDC-0941 for a 24 and 48 h period. Thereafter floating and attached cells were collected and stained with 7-AAD and Annexin V antibodies (Annexin V-PE detection kit I, BD Bioscience) and analysed by flow cytometry (Navios, Beckman Coulter). Proliferation was traced by CFSE staining and apoptosis was detected by combined 7AAD/Annexin V staining and calculated in percent of control.

### Cell migration assay

The *in vitro* scratch assay was performed as described by Liang et al. [48]. Briefly, Daoy (5 × 10^5^/6 well) cells were plated in 12-well cell culture dishes. The cells were allowed to adhere and spread for 12 h at 37°C. The confluent monolayer was scratched in a straight line with a p200 pipette tip. The debris was removed and the cells were then incubated with different concentrations of GDC-0941. The vehicle DMSO served as control. After 12 and 24 h of treatment, migration of cells into the scratch was photographed at 10× magnification (Eclipse TiS inverted microscope attached to a CCD monochrome camera DS 2M, Nikon). The distance of migration was analyzed by means of NIS-Elements Imaging Software (Nikon).

### Cell cycle assay

Daoy (2 × 10^5^/6 well) and MEB-Med-8A (3 × 10^5^/well) cells respectively were plated in 6-well cell culture dishes. After 48 h of treatment with GDC-0941 the cells were exposed to 16 nM Hoechst 33342 and incubated for 45min at 37°C. Both floating and attached cells were harvested and analyzed by flow cytometry (Navious, Beckman Coulter). Dead cells were stained by propidium iodide. After gating on live cells, single cells were gated using width and area parameters from Hoechst 33342. The area parameter histogram was used to determine the percentage of cells in G_1_, S and G_2_M phases.

### Immunoblotting analysis

Medulloblastoma cell lines were lysed in RIPA buffer supplemented with protease and phosphatase inhibitors (Roche). Subsequently 25 μg of protein were separated by SDS-polyacrylamide gel electrophoresis and transferred to nitrocellulose membranes (BioRad). The membranes were blocked for 1 h at RT in Tris-buffered saline containing 0.1% tween-20 (TBST) supplemented with 5% BSA. Thereafter, the membranes were incubated with the primary antibodies (1/1000) overnight at 4°C and subsequently with the respective secondary antibody (1/10000) for 1 h at room temperature. Immunoreactivity was detected by chemiluminescence and quantified by means of a ChemiDoc XRS Imaging System (Bio-Rad).

### Colony formation assay

The cell lines Daoy (200 cells/6 well) and MEB-Med-8A (1000 cells/6 well) were plated in six well cell culture dishes. The cells were allowed to adhere and were incubated for 12 h at 37°C. Thereafter the cells were exposed to 1 and 10 μM of GDC-0941 respectively. After 48 h the cells were washed with standard medium to remove the inhibitor and cultured for another week. Colony numbers and colony size were assessed by IMAGEJ. Particles smaller than 20 pixel^2^ were excluded from the analysis since these represented mainly staining artefacts, cell detritus or non-proliferating single cells.

### Lentiviral particles and stable cell lines

Lentivirale particles were generated by co-transfection of HEK293T Lenti-X cells (Clontech) with packaging plasmids (pMD2.G, pMDLg/pRRE, pRSV-REV, Addgen) and the lentiviral transfervector (pLenti-III-UbC-Luc2, Applied Biological Materials Inc.) expressing Luciferase 2. After 48 and 72 hours the supernatants were pooled, filtered through a 45 um filter and ultracentrifuged at 87076 g 4°C for 2 h. The virus titers were determined by HIV p24 antigen test (Elecsys, Roche Diagnostics). MEB-Med-8A cells were transduced with virus particles (MOI 5) and thereafter selected with puromycin and validated for homogenous luciferase activity (Invitrogen).

### Orthotopic transplantation and tumor formation

Immunocompromised NSG mice were used for transplantation. The animals were bred and housed in a specific pathogen-free animal facility at the house for experimental therapy of the University of Bonn. All experiments were conducted according to protocols approved by the institutional animal use and care committee of Northrhine Westphalia (Germany). To establish intracranial tumors, medulloblastoma cells were resuspended in PBS and injected perpendicular to the cranial surface via 5 μL pst2 Hamilton 7105N syringe into the right cerebellar hemisphere (1 mm to the right of the midline, 1 mm posterior to the coronal suture, and 3 mm deep) of 5–9 week old anesthetized NSG mice. fixed in a stereotaxic frame. After transplantation the animals were monitored daily and sacrificed when symptoms of tumor growth occured. All procedures were in strict accordance with the University of Bonn Medical Center Policy on the Use and Care of Laboratory Animals (University of Bonn Medical Center Policy and Welfare Committee, Document ID: 87-51.04.2011.A033).

### *In vivo* bioluminescent imaging

For the bioluminescent imaging the mice were anesthetized by ketamine/xylazine. injected intraperitoneally with 125 mg/kg D-luciferin. 15 min after injection the animals were imaged using a IVIS 200 imaging station (Caliper Life Sciences). Regions of interest were defined using living image software, and the total photons/s/sr/cm^2^ (photons per second per steradian per square cm) were recorded weekly to monitor tumor growth and therapy response. To determine the growth rate of the tumor the gain in bioluminescence per week was calculated.

### *In vivo* inhibitor treatment

To study effects of the PI3K inhibitor GDC-0941 on tumor growth *in vivo* we transplanted 2 × 10^4^ MEB-Med-8A cells into the cerebellum of NSG mice. Seven days after transplantation the mice were randomly separated into two groups. Group 1 was given the vehicle EL-ethanol (60:40; cremophor EL, 95% ethyl alcohol, Sigma) while group 2 was exposed to 100 mg/kg GDC-0941. The drug was administered once daily by oral gauge. Event-free survival was defined as the time from transplantation until symptom onset.

### Statistical analysis

The two-sided Student's *t* test was applied to determine statistically significant differences between two groups. For *in vivo* tumor growth and survival the log-rank Test (Mantel-Cox) and Wilcoxon signed rank test respectively were applied to determine statistically significant differences. *p* < 0.05 (*) was considered as statistically significant. The values stated within text and figures are mean ± standard deviation.

## SUPPLEMENTARY FIGURES



## References

[1] Gajjar A, Chintagumpala M, Ashley D, Kellie S, Kun LE, Merchant TE, Woo S, Wheeler G, Ahern V, Krasin MJ, Fouladi M, Broniscer A, Krance R, Hale GA, Stewart CF, Dauser R (2006). Risk-adapted craniospinal radiotherapy followed by high-dose chemotherapy and stem-cell rescue in children with newly diagnosed medulloblastoma : long-term results from a prospective, multicentre trial. Lancet Oncol.

[2] Mabbott DJ, Spiegler BJ, Greenberg ML, Rutka JT, Hyder DJ, Bouffet E (2005). Serial evaluation of academic and behavioral outcome after treatment with cranial radiation in childhood. J Clin Oncol.

[3] Louis DN, Ohgaki H, Wiestler OD, Cavenee WK, Burger PC, Jouvet A, Scheithauer BW, Kleihues P (2007). The 2007 WHO classification of tumours of the central nervous system. Acta Neuropathol.

[4] Northcott PA, Shih DJ, Peacock J, Garzia L, Sorana Morrissy A, Zichner T, Stutz AM, Korshunov A, Reimand J, Schumacher SE, Beroukhim R, Ellison DW, Marshall CR, Lionel AC, Mack S, Dubuc A (2012). Subgroup-specific structural variation across 1,000 medulloblastoma genomes. Nature.

[5] Hartmann W, Digon-Sontgerath B, Koch A, Waha A, Endl E, Dani I, Denkhaus D, Goodyer CG, Sorensen N, Wiestler OD, Pietsch T (2006). Phosphatidylinositol 3′-kinase/AKT signaling is activated in medulloblastoma cell proliferation and is associated with reduced expression of PTEN. Clin Cancer Res.

[6] Baryawno N, Sveinbjornsson B, Eksborg S, Chen CS, Kogner P, Johnsen JI (2010). Small-molecule inhibitors of phosphatidylinositol 3-kinase/Akt signaling inhibit Wnt/beta-catenin pathway cross-talk and suppress medulloblastoma growth. Cancer Res.

[7] Guerreiro AS, Fattet S, Fischer B, Shalaby T, Jackson SP, Schoenwaelder SM, Grotzer MA, Delattre O, Arcaro A (2008). Targeting the PI3K p110alpha isoform inhibits medulloblastoma proliferation, chemoresistance, and migration. Clin Cancer Res.

[8] Blom T, Roselli A, Hayry V, Tynninen O, Wartiovaara K, Korja M, Nordfors K, Haapasalo H, Nupponen NN (2010). Amplification and overexpression of KIT, PDGFRA, and VEGFR2 in medulloblastomas and primitive neuroectodermal tumors. J Neurooncol.

[9] Liu W, Zhang S, Zhang L, Cui Q, Wang J, Gui T, Pang Q (2014). A prognostic analysis of pediatrics central nervous system small cell tumors: evaluation of EGFR family gene amplification and overexpression. Diagnostic pathology.

[10] Craveiro RB, Ehrhardt M, Holst MI, Pietsch T, Dilloo D (2014). In comparative analysis of multi-kinase inhibitors for targeted medulloblastoma therapy pazopanib exhibits promising *in vitro* and *in vivo* efficacy. Oncotarget.

[11] MacDonald TJ, Brown KM, LaFleur B, Peterson K, Lawlor C, Chen Y, Packer RJ, Cogen P, Stephan DA (2001). Expression profiling of medulloblastoma: PDGFRA and the RAS/MAPK pathway as therapeutic targets for metastatic disease. Nature genetics.

[12] Torbett NE, Luna-Moran A, Knight ZA, Houk A, Moasser M, Weiss W, Shokat KM, Stokoe D (2008). A chemical screen in diverse breast cancer cell lines reveals genetic enhancers and suppressors of sensitivity to PI3K isoform-selective inhibition. Biochem J.

[13] Zheng Z, Amran SI, Thompson PE, Jennings IG (2011). Isoform-selective inhibition of phosphoinositide 3-kinase: identification of a new region of nonconserved amino acids critical for p110alpha inhibition. Mol Pharmacol.

[14] Ihle NT, Powis G (2010). Inhibitors of phosphatidylinositol-3-kinase in cancer therapy. Mol Aspects Med.

[15] Bain J, Plater L, Elliott M, Shpiro N, Hastie CJ, McLauchlan H, Klevernic I, Arthur JS, Alessi DR, Cohen P (2007). The selectivity of protein kinase inhibitors: a further update. Biochem J.

[16] Gharbi SI, Zvelebil MJ, Shuttleworth SJ, Hancox T, Saghir N, Timms JF, Waterfield MD (2007). Exploring the specificity of the PI3K family inhibitor LY294002. Biochem J.

[17] Ihle NT, Powis G (2009). Take your PIK: phosphatidylinositol 3-kinase inhibitors race through the clinic and toward cancer therapy. Mol Cancer Ther.

[18] Folkes AJ, Ahmadi K, Alderton WK, Alix S, Baker SJ, Box G, Chuckowree IS, Clarke PA, Depledge P, Eccles SA, Friedman LS, Hayes A, Hancox TC, Kugendradas A, Lensun L, Moore P (2008). The identification of 2-(1H-indazol-4-yl)-6-(4-methanesulfonyl-piperazin-1-ylmethyl)-4-morpholin-4-yl-thieno[3,2-d]pyrimidine (GDC-0941) as a potent, selective, orally bioavailable inhibitor of class I PI3 kinase for the treatment of cancer. Journal of medicinal chemistry.

[19] Besse B, Soria J, Gomez-Roca C, Ware JA, Adjei AA, Dy GK, Shankar GR, Brachmann K, Groen HJ (2011). A phase Ib study to evaluate the PI3-kinase inhibitor GDC-0941 with paclitaxel (P) and carboplatin (C), with and without bevacizumab (BEV), in patients with advanced non-small cell lung cancer (NSCLC). J Clin Oncol.

[20] Moreno Garcia, Baird RD, K S, Basu JB, Tunariu N, Blanco M, Cassier PA, Pedersen JV, Puglisi M, Sarker D, Papadatos-Pastos DA, Omlin G, Biondo A, Ware JA, Koeppen H, Levy GG (2011). A phase I study evaluating GDC-0941, an oral phosphoinositide-3 kinase (PI3K) inhibitor, in patients with advanced solid tumors or multiple myeloma. J Clin Oncol.

[21] Von Hoff DD, LoRusso P, Demetri GD, Weiss GJ, Shapiro G, Ramanathan RK, Ware JA, Raja R, Jin J, Levy GG, Mazina KE, Wagner AJ (2011). A phase I dose-escalation study to evaluate GDC-0941, a pan-PI3K inhibitor, administered QD or BID in patients with advanced or metastatic solid tumors. J Clin Oncol.

[22] Von Hoff DD, LoRusso P, Tibes R, Shapiro G, Weiss GJ, Ware J, Fredrickson AJ, Mazina KE, Levy GG, Wagner AJ (2010). A first-in-human phase I study to evaluate the pan-PI3K inhibitor GDC-0941 administered QD or BID in patients with advanced solid tumors. J Clin Oncol.

[23] Calabrese CM, Gaber WM, Kilmar J, Fuller C, Allen M, Gilbertson RJ Autofluorescent mouse models of human medulloblastoma. Proc Amer Assoc Cancer Res.

[24] Friedman HS, Burger PC, Bigner SH, Trojanowski JQ, Brodeur GM, He XM, Wikstrand CJ, Kurtzberg J, Berens ME, Halperin EC (1988). Phenotypic and genotypic analysis of a human medulloblastoma cell line and transplantable xenograft (D341 Med) demonstrating amplification of c-myc. Am J Pathol.

[25] Rosen ST (2002). Clinically relevant resistance in cancer chemotherapy.

[26] Bodey BS, Kaiser HE, Siegel SE (2004). Molecular Markers of Brain Tumor Cells.

[27] Briggs KJ, Corcoran-Schwartz IM, Zhang W, Harcke T, Devereux WL, Baylin SB, Eberhart CG, Watkins DN (2008). Cooperation between the Hic1 and Ptch1 tumor suppressors in medulloblastoma. Genes Dev.

[28] Yokota N, Mainprize TG, Taylor MD, Kohata T, Loreto M, Ueda S, Dura W, Grajkowska W, Kuo JS, Rutka JT (2004). Identification of differentially expressed and developmentally regulated genes in medulloblastoma using suppression subtraction hybridization. Oncogene.

[29] Jacobsen PF, Jenkyn DJ, Papadimitriou JM (1985). Establishment of a human medulloblastoma cell line and its heterotransplantation into nude mice. J Neuropathol Exp Neurol.

[30] Calabrese C, Poppleton H, Kocak M, Hogg TL, Fuller C, Hamner B, Oh EY, Gaber MW, Finklestein D, Allen M, Frank A, Bayazitov IT, Zakharenko SS, Gajjar A, Davidoff A, Gilbertson RJ (2007). A perivascular niche for brain tumor stem cells. Cancer Cell.

[31] Liang J, Slingerland JM (2003). Multiple roles of the PI3K/PKB (Akt) pathway in cell cycle progression. Cell Cycle.

[32] Gong C, Liao H, Wang J, Lin Y, Qi J, Qin L, Tian LQ, Guo FJ (2012). LY294002 induces G0/G1 cell cycle arrest and apoptosis of cancer stem-like cells from human osteosarcoma via down-regulation of PI3K activity. Asian Pac J Cancer Prev.

[33] Vogt PK, Hart JR (2011). PI3K and STAT3: a new alliance. Cancer Discov.

[34] Teicher BA (2006). Tumor models for efficacy determination. Mol Cancer Ther.

[35] Salphati L, Heffron TP, Alicke B, Nishimura M, Barck K, Carano RA, Cheong J, Edgar KA, Greve J, Kharbanda S, Koeppen H, Lau S, Lee LB, Pang J, Plise EG, Pokorny JL (2012). Targeting the PI3K pathway in the brain—efficacy of a PI3K inhibitor optimized to cross the blood-brain barrier. Clin Cancer Res.

[36] Bacolod MD, Lin SM, Johnson SP, Bullock NS, Colvin M, Bigner DD, Friedman HS (2008). The gene expression profiles of medulloblastoma cell lines resistant to preactivated cyclophosphamide. Curr Cancer Drug Targets.

[37] Friedman HS, Dolan ME, Pegg AE, Marcelli S, Keir S, Catino JJ, Bigner DD, Schold SC (1995). Activity of temozolomide in the treatment of central nervous system tumor xenografts. Cancer Res.

[38] Metcalfe C, Alicke B, Crow A, Lamoureux M, Dijkgraaf GJ, Peale F, Gould SE, de Sauvage FJ (2013). PTEN loss mitigates the response of medulloblastoma to Hedgehog pathway inhibition. Cancer Res.

[39] Hart JR, Vogt PK (2011). Phosphorylation of AKT: a mutational analysis. Oncotarget.

[40] Graupera M, Potente M (2013). Regulation of angiogenesis by PI3K signaling networks. Exp Cell Res.

[41] Manning BD, Cantley LC (2007). AKT/PKB signaling: navigating downstream. Cell.

[42] Hay N, Sonenberg N (2004). Upstream and downstream of mTOR. Genes Dev.

[43] Yu H, Jove R (2004). The STATs of cancer—new molecular targets come of age. Nat Rev Cancer.

[44] Yu H, Pardoll D, Jove R (2009). STATs in cancer inflammation and immunity: a leading role for STAT3. Nat Rev Cancer.

[45] Jackson C, Ruzevick J, Amin AG, Lim M (2012). Potential role for STAT3 inhibitors in glioblastoma. Neurosurg Clin N Am.

[46] Cattaneo E, Magrassi L, De-Fraja C, Conti L, Di Gennaro I, Butti G, Govoni S (1998). Variations in the levels of the JAK/STAT and ShcA proteins in human brain tumors. Anticancer Res.

[47] Yang F, Van Meter TE, Buettner R, Hedvat M, Liang W, Kowolik CM, Mepani N, Mirosevich J, Nam S, Chen MY, Tye G, Kirschbaum M, Jove R (2008). Sorafenib inhibits signal transducer and activator of transcription 3 signaling associated with growth arrest and apoptosis of medulloblastomas. Mol Cancer Ther.

[48] Liang CC, Park AY, Guan JL (2007). *In vitro* scratch assay: a convenient and inexpensive method for analysis of cell migration *in vitro*. Nat Protoc.

