# Supplementary material for: The PI3K inhibitor GDC-0941 displays promising in vitro and in vivo efficacy for targeted medulloblastoma therapy
Source: Oncotarget. 2014 Dec 6;6(2):802–13. doi: 10.18632/oncotarget.2742 (PMC4359256; doi:10.18632/oncotarget.2742)
Supplement: Supplementary file 1 [file oncotarget-06-802-s001.pdf]

SUPPLEMENTARY FIGURES

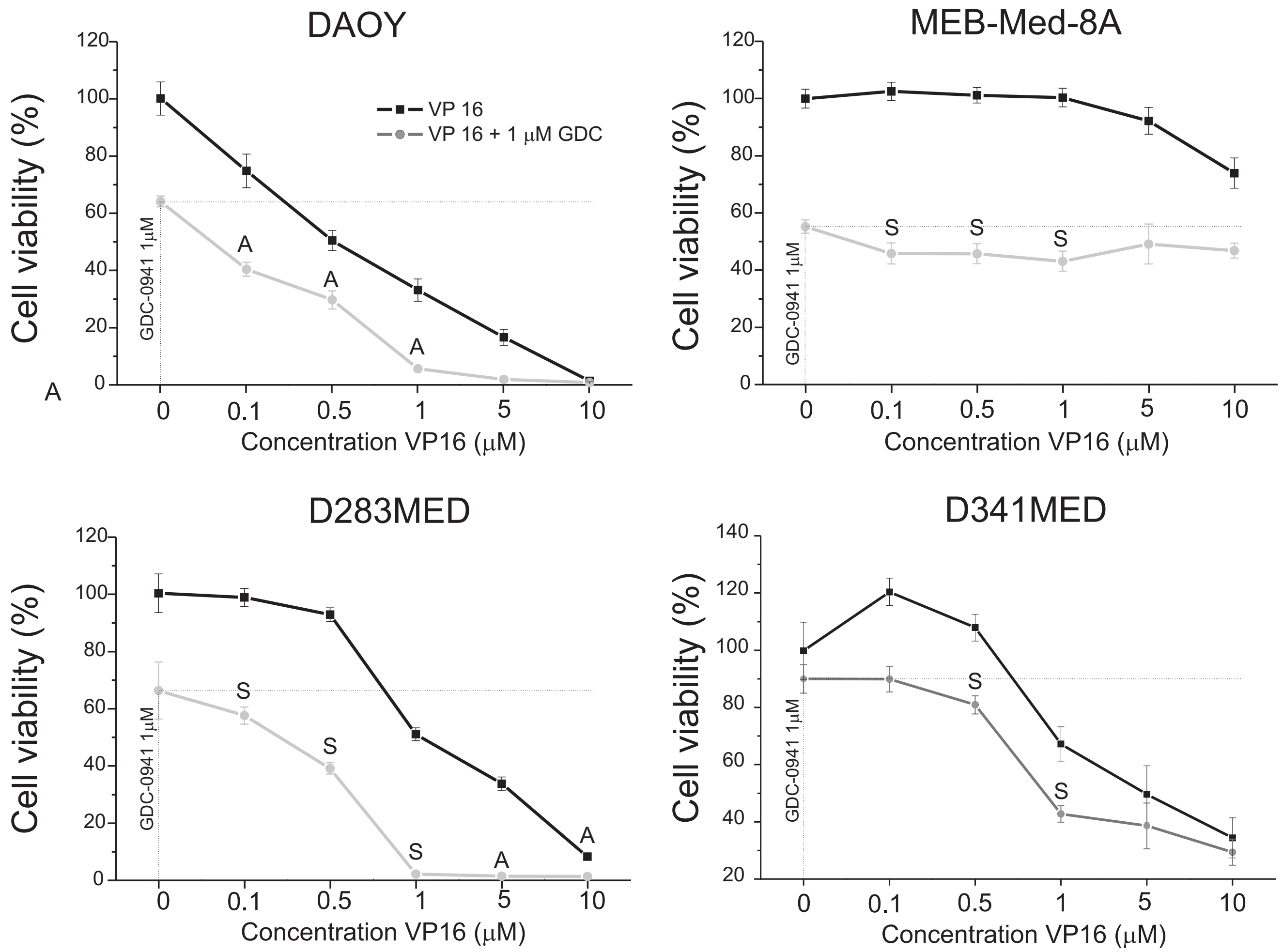

Supplementary Figure 1: GDC-0941 synergizes with etoposide a standard chemotherapeutic in pediatric medulloblastoma treatment. MEB-Med-8A, D283 Med, Daoy and D341 Med were treated in combination with increasing concentrations of etoposide (0.5, 1, 5 and 10 μM) and 1 μM of GDC-0941. The vehicle DMSO served as control. Of note, 1–2 μM etoposide corresponds to cerebrospinalfluid levels of etoposide determined in treated patients. After 48 h drug exposure the cell viability was assessed by means of the MTS assay. We determined if GDC-0941 synergizes with etoposide and leads to greater decrease in cell viability than the sum of the compounds individual effects. For the combinational treatment curve "S" indicates synergistic effects, while "A" marks additive effects of the drugs. Each experiment was performed in triplicates and repeated four times.

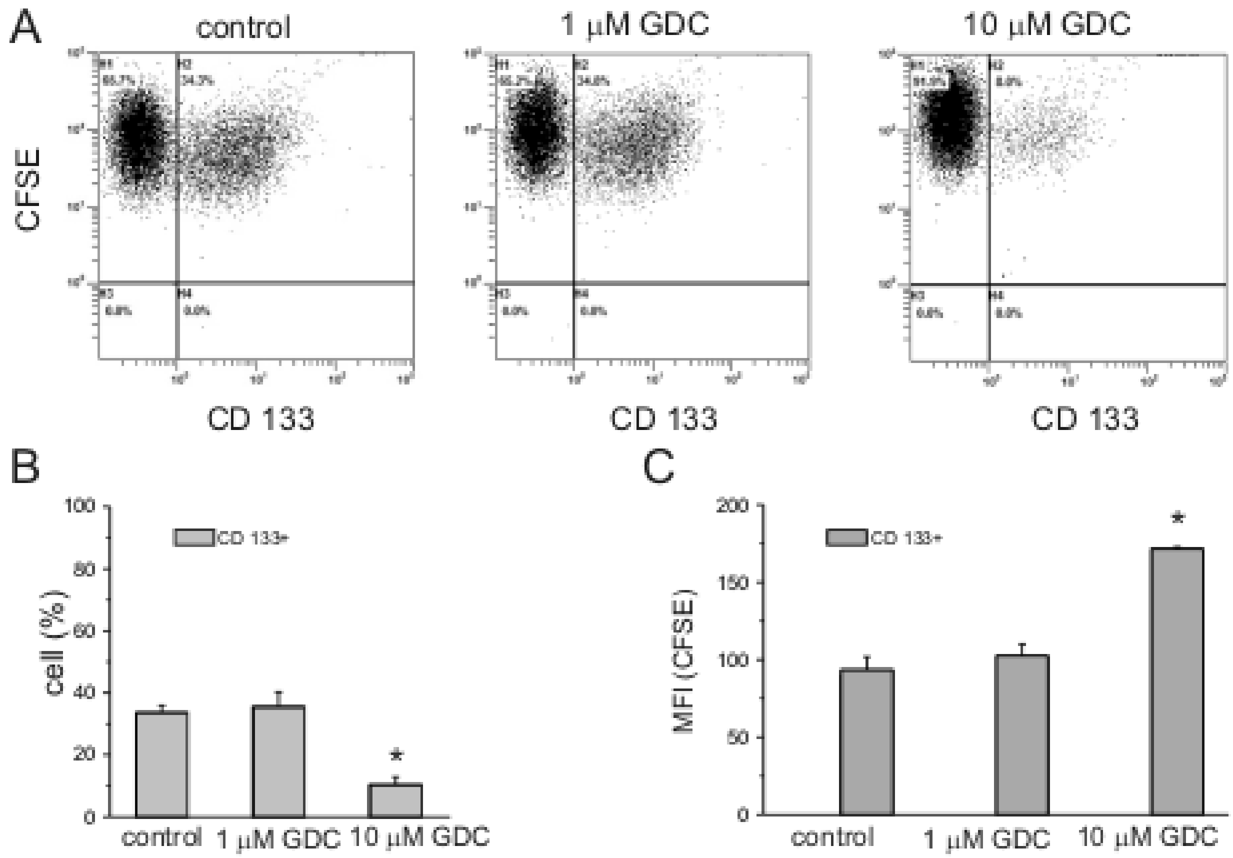

Supplementary Figure 2: GDC-0941 inhibits the proliferation of CD133-positive MB cells. When screening the investigated MB cell lines for expression of the cancer stem cell marker CD133, only in D283 Med we could detect substantial expression of CD133. Therefore, we chose this cell line to analyze whether GDC-0941 would also affect the proliferation of CD133 expressing cells. For this purpose D283 Med were CFSE stained and exposed to 1 and 10 µM of GDC-0941. The vehicle DMSO served as control. After 48 h of treatment the proliferation of the CD133-positve cell population was traced via flow cytometry. Statistically significant differences are marked by an asterisk (*$p < 0.05$). The data shown represent four independent experiments.
